# A Bilayer SnO_2_/MoS_2_-Coated Evanescent Wave Fiber Optic Sensor for Acetone Detection—An Experimental Study

**DOI:** 10.3390/bios12090734

**Published:** 2022-09-07

**Authors:** A. Prasanth, Selamawit Getachew, Tseganesh Shewa, M. Velumani, S. R. Meher, Z. C. Alex

**Affiliations:** 1 Department of Sensor and Biomedical Technology, School of Electronics Engineering, Vellore Institute of Technology, Vellore 632014, India; 2 Department of ECE, Madanapalle Institute of Technology and Science, Madanapalle 517325, India; 3 Department of Physics, School of Advanced Sciences, Vellore Institute of Technology, Vellore 632014, India

**Keywords:** fiber optic sensor, evanescent wave, acetone, SnO_2_, MoS_2_

## Abstract

The need for sensors that measure the acetone content of exhaled breath for diabetes severity has recently increased. Clinical researchers have reported less than 0.8 ppm acetone concentration in the exhaled breath of an average individual, while that for a diabetic patient is higher than 1.8 ppm. This work reports the development of two sets of evanescent wave-based fiber optic sensor coated with SnO_2_ thin film and bilayer of SnO_2_/MoS_2_ to detect different acetone concentrations (0–250 ppm). In each set, we have studied the effect of clad thickness (chemical etch time 5min, 10 min, 15 min, 25 min, 40 min, and complete clad removal) to optimize the clad thickness for a better response. In Set 1, SnO_2_ thin film was used as the sensing layer, while in Set 2 a bilayer of SnO_2_ thin film/ MoS_2_ was used. Enhanced sensor response of ~23.5% is observed in the Set 2 probe with a response and recovery time of ~14 s/~17 s. A SnO_2_/MoS_2_-coated sensor prototype is developed using LEDs of different wavelength and intensity detector; its potential to detect different concentrations of acetone is tested. X-ray Diffraction (XRD), Scanning Electron Microscope (SEM), Ultraviolet (UV) Spectroscopy, and Ellipsometry were used to study the structural, morphological and optical properties of the sensing layers. The present study indicates that the SnO_2_/MoS_2_-coated sensor has the potential to create a handheld sensor system for monitoring diabetes.

## 1. Introduction

Diabetes is a chronic disease that occurs when the body cannot use blood sugar (glucose) properly because the body does not make sufficient insulin; therefore, insulin must be injected regularly every day to stay alive. The body cannot efficiently utilize glucose for energy if there is inadequate insulin [1]. This leads to the release of hormones that break down fat as fuel producing acids known as ketone. The process of creating ketone is called ketogenesis. Aceto-acetate is generated from acetyl co-enzyme A that spontaneously decarboxylates to acetone; this can be used as a non-invasive biomarker for identifying diabetes [2]. The invasive way of detecting diabetes is highly uncomfortable for patients. Pricking the side of the finger with a needle and taking blood may create bruised skin, leading to infection. Due to this, many people are afraid of invasive blood testing methods; this causes diabetes to be unchecked and leads to a high mortality rate.

Exhaled breath analysis has become more prominent as analytical methods and nanotechnology have advanced. It has caught the interest of researchers owing to its potential for disease diagnosis and precision treatment. This study has the potential to replace standard blood tests, which are intrusive and painful; the proposed work is non-invasive, cost-effective, can be done in real-time and offers qualitative/quantitative illness diagnosis. Non-invasive therapies are therefore in great demand. Analyzing VOCs in exhaled breath monitors metabolic conditions, medication adherence, and disease diagnosis [3]. In addition to 78% nitrogen (N_2_), 16% oxygen (O_2_), 5% carbon dioxide (CO_2_), and 5% hydrogen (H_2_), human exhalation contains different compounds such as acetone, ethanol, acetophenone, isoprene, ethane, pentane, and methane [4]. As inhaled air travels down to the alveoli in the lungs, these metabolic products are dispersed in the inhaled air and rejected as exhaled air. Thus, an endogenous metabolic activity is present in the exhaled air. This can be used as an essential tool for recognizing disorders and tracking one’s health [5,6,7]. Finding a specific VOC with a low ppm/ppb concentration might be difficult. A recent clinical study has demonstrated that although an average person’s exhaled breath has an acetone content of less than 0.8 ppm, diabetics have an acetone concentration of more than 1.8 ppm. This necessitates the need for sensors that check the amount of acetone in exhaled breath for identifying the severity of diabetes. Due to its non-invasive nature and capability of real-time monitoring, breath analysis to identify the concentration of acetone has gained a lot of attention as an effective method for investigating diabetes. Although it is currently challenging, exhaled breath compounds can be analysed for medical purposes [8,9]. It is crucial to create straightforward portable devices capable of providing consistent and ongoing real-time monitoring for breath markers like acetone [10].

Much recent research have shown interest in fiber optic-based sensors because of their high sensitivity, tolerance to electromagnetic interference, compact size, and low weight [11]. Fiber optic-based breath analysers can be developed using the sensing mechanism based on clad modification approach. It includes varying the clad thickness and depositing nanoparticle layers as the sensing region. B. Renganathan et al. [12] utilized Zinc oxide (ZnO) nanoparticles as the sensing layer in clad modification approach, while Zn_3_(VO_4_)_2_ nanoparticles was used by M. Subramanian et al. [13]. Despite being inexpensive, simple to make, and very sensitive, nanoparticle-based sensors have certain drawbackslike slow sensor response and reaction time. Due to the limitations of nanoparticle-coated optical sensors, we have employed a thin film-coated fiber optic sensor in our previous work [14]. The sensor utilized three single layer materials, such as, ZnO, Aluminum doped Zinc Oxide (AZO), and Tin Oxide (SnO_2_). Of these, the SnO_2_-coated sensor showed maximum response of 21.2% for IPA (250 ppm) and 13.9% for acetone (250 ppm) due to its favorable optical conditions [14]. R. Bhardwaj et al. [15] used MoS_2_ as the sensing material for lower concentrations of acetone. Along with SnO_2_, MoS_2_ has suitable optical conditions for evanescent sensing principle. MoS_2_ is notable for its vertically stacked layers generated by covalently bound Mo-S atoms [16]. MoS_2_ is an ideal material for gas sensing because each neighbouring layer is held together by weak Van der waals forces, allowing gas molecules to penetrate and diffuse easily between the layers [17]. H. Chen et al. [18] has used bilayered SnO_2_-MoS_2_ as the sensing material and achieved better sensing response over single-layered sensors. Based on these research outcomes, we chose MoS_2_ as the second layer over the SnO_2_ layer to improve its sensing behavior and make the probe more sensitive to acetone. 

This could be the first experimental work usingevanescent wave-based sensor coated with SnO_2_-MoS_2_ bilayers for acetone detection. Moreover, the effect of clad thickness on acetone concentration is investigated in terms of intensity variation. Two sets of acetone sensors were fabricated with each set containing six sensor probes. Set 1 comprises of SnO_2_ thin film as the sensing layer with different clad thicknesses (Sensor probe A.1, A.2, A.3, A.4, A.5 and A.6), and set 2 comprises of SnO_2_/MoS_2_ bilayer as the sensing region (Sensor probe B.1, B.2, B.3, B.4, B.5 and B.6). The sensor responses of the proposed probes were measured in terms of intensity variation towards acetone concentrations varying from 0 ppm to 250 ppm.

## 2. Preparation of Sensor Probes

**Figure 1:**
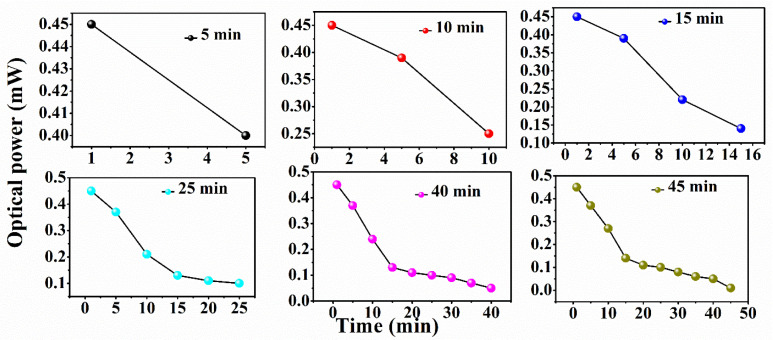
Power drop measurement was performed by employing the power measuring experimental setup to observe the interaction of diluted 40% HF with plastic clad. The decrease in power of the suggested sensor probes was tested across various clad etching time periods of 5 min, 10 min, 15 min, 25 min, 40 min, and 45 min.

**Figure 2:**
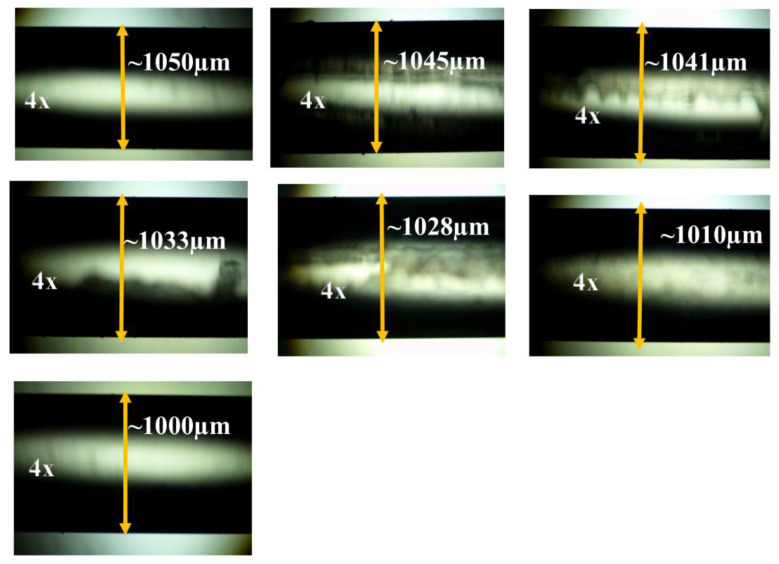
The decrease in plastic clad subject to various time scales is assessed using microscope images, whilst the rise in chemical etching time scale reduces the clad part. Microscopic pictures (4×) demonstrate the removal of plastic clads from optical fibers by a chemical etching technique using hydrofluoric acid (40%) diluted with distilled water. The clad-core portions are measured at 1050 µm, 1045 µm, 1041 µm, 1033 µm, 1028 µm, 1010 µm, and 1000 µm.

The sensor probes are developed using plastic-clad silica (PCS) core optical fiber (0.39 N.A) with a core diameter of 1000 µm (FT1000EMT, Thorlabs, Newton, NJ, USA). To prepare the sensing region, 2 cm of the outer jacket of the optical fiber is removed mechanically using a surgical blade. Then, the different thicknesses of clad layer are etched using the chemical etching technique. Hydrofluoric Acid (HF 40%) is used to removes the clad portion of the optical fiber. After removal of the outer jacket, the optical fiber is immersed in the 5 mL HF diluted with 20 mL distilled water. The different etching rates of the clad layer is achieved by engaging the optical fiber in different periods, namely, 5 min, 10 min, 15 min, 25 min, 40 min, and complete clad removal. To confirm chemical etching, one end of the fiber is connected to the light source and the other with an optical power meter (PM100USB, Thorlabs). The output power of the immersed optical fiber is continuously monitored. The results exhibit that power is sequentially reduced with time, as shown in Figure 1. The thickness of the clad layer of the optical fiber is measured using microscopic analysis, as shown in Figure 2. All six etched optical fibers were deposited with the SnO_2_ thin film layer using Radio Frequency (RF) sputtering technique. A deposition pressure of 25 × 10^−3^ mbar and base pressure of 3 × 10^−5^ mbar with RF power of 50 W were applied for deposition of the SnO_2_ layer. To achieve uniformity of the SnO_2_ layer, a rotating setup that continuously rotated during deposition was developed inside the chamber.

**Figure 3:**
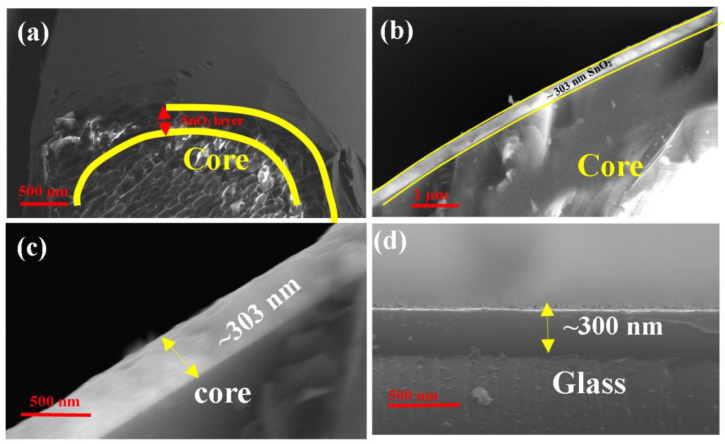
Cross-sectional SEM images of (**a**) SnO_2_ thin film coated over optical fiber, (**b**) magnified image of optical fiber, (**c**) deep magnified image of optical fiber, (**d**) glass substrate using RF Magnetron sputtering technique based on the deposition condition in Section 2.

Further, the thickness of the SnO_2_ layer is measured using a glass substrate (1 cm × 1 cm) that was fixed inside the deposition chamber along with the optical fiber. The thickness of SnO_2_ coated over the glass substrate is measured using an ellipsometer (J.A. Wollam, Alpha SE). The ellipsometer results showed that the thickness of the SnO_2_ layer is ~300 nm; this is also confirmed in cross sectional SEM image (shown in Figure 3d).

**Figure 4:**
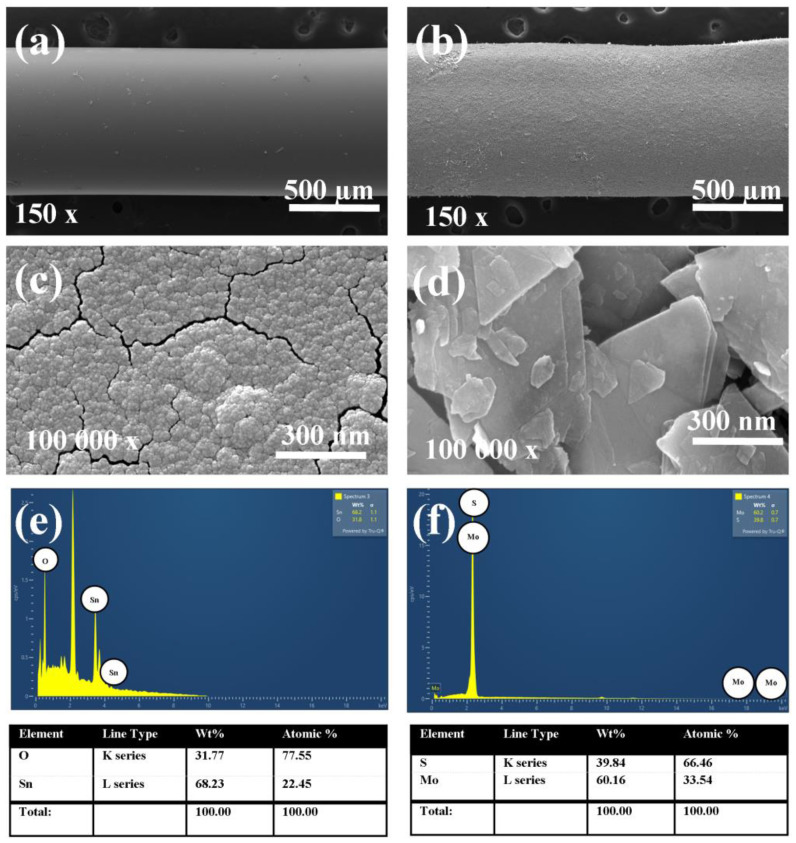
SEM images of the proposed sensor probe coated with (**a**) SnO_2_ thin film, (**b**) SnO_2_/MoS_2_ layer, magnified surface images of (**c**) SnO_2_ thin film, and (**d**) SnO_2_/MoS_2_ layer. The presence of components on the surface is confirmed by EDS images (**e**,**f**).

Moreover, to confirm the thickness of SnO_2_ layer over the optical fiber, the cross-sectional analysis of optical fiber was carried out in different scales as shown in Figure 3a–c. The result indicates that thickness is around ~303 nm with an error of ±3 nm(ellipsometer value). The same deposition time and pressure are maintained for all the six optical fibers. The uniformity of the SnO_2_ layer and its elemental composition are shown in Figure 4a,c,e. After the deposition of SnO_2_, the MoS_2_ layer is coated as the second layer using the dip-coating technique. The structure and thickness of the MoS_2_ layer above the SnO_2_ layer, as well as its elemental compositions, are determined by SEM images, as shown in Figure 4b,d,f. The magnified views of the MoS_2_ layer show that the structure is non-homogeneous with gaps between the MoS_2_ particles.

## 3. Structural Properties of SnO_2_ and MoS_2_Layer

**Figure 5:**
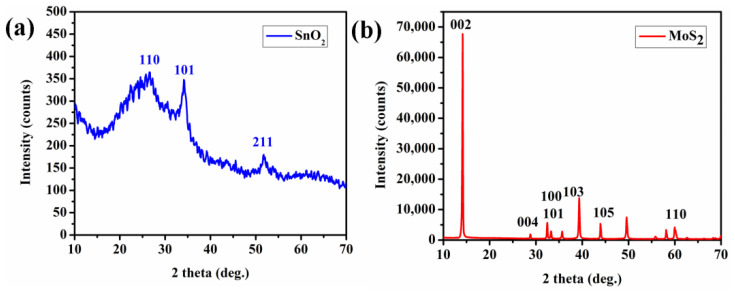
X-ray diffraction patterns to study the structural properties of thin films of (**a**) SnO_2_layer desposited by sputtering technique, and (**b**) MoS_2_layer by dip coating technique, on glass substrates.

The XRD pattern of SnO_2_ thin film and MoS_2_ layer is observed in the range of 20–70 °C (shown in Figure 5a,b). The XRD pattern of SnO_2_ thin films indicates significant peaks at (110), (101), and (211) counts, resulting in a polycrystalline structure with a tetragonal phase (shown in Figure 5a). The diffraction peaks in Figure 5b located at 2θ values of 33°, 40°, 50°, and 59° correspond to (100), (103), (105), and (110) planes of the hexagonal phase of MoS_2_, respectively.

## 4. Optical Properties of SnO_2_ and MoS_2_Layer

**Figure 6:**
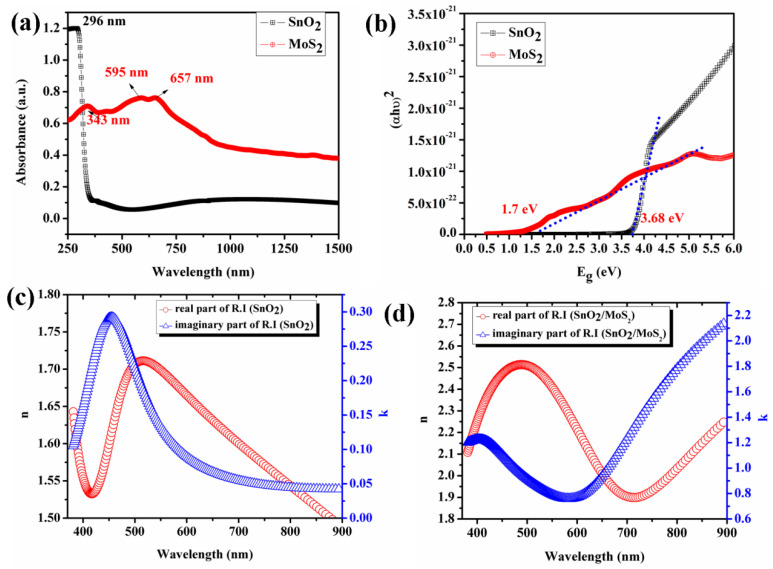
(**a**) UV-Vis spectra ofSnO_2_ thin film and MoS_2_, (**b**) Tauc’s plot of SnO_2_ thin film and MoS_2_ coated on the glass substrate (2.5 cm × 2.5cm), and optical constants of (**c**) SnO_2_ and (**d**) MoS_2_.

The optical property of SnO_2_ thin film and MoS_2_ layer coated glass substrate was investigated using UV–visible spectroscopy and ellipsometer. To analyze the optical properties of the sensing layers (namely, SnO_2_ and SnO_2_/MoS_2_ layer), the coating is done on the glass substrate under the deposition condition discussed in Section 2. The optical properties of SnO_2_ thin film and MoS_2_ layer coated glass substrate were investigated using UV–visible spectroscopy and ellipsometer. The prepared SnO_2_ thin film shows a sharp absorption edge around the UV range (~296 nm), as shown in Figure 6a, while MoS_2_ reveals the absorption band near 343 nm, 595 nm, and 657 nm. Further, the optical band gap (E_g_) of SnO_2_ and MoS_2_ were obtained using Tauc’s equation:

(αhν)^2^ = A(hν − Eg) (1)

where A is a proportionality constant, ‘hν’ is the incident photon energy, ‘α’ is the absorption coefficient, and ‘Eg’ is the optical bandgap. Figure 6b shows the plot of (αhν)^2^ vs. hν; it is seen that the optical band gap of SnO_2_ thin film is 3.67 eV and MoS_2_ is 1.7 eV. The optical constants n and k play a significant role in the evanescent wave-based sensors. The real part of the sensing layer’s refractive index resolves the internal reflection type [19]. When the real part of refractive index of the sensing layer (n_clad_) is higher than the core’s refractive index (n_c_), the condition of partial internal reflection occurs [12]. Figure 6c,d shows the optical constants of the sensing layer SnO_2_ and bilayer SnO_2_/MoS_2,_ whose real part of refractive index is higher than the core’s refractive index of 1.453 (SiO_2_) [14]. This confirms that the proposed system works under the condition of partial internal reflection. The refractive index condition of the film increases significantly after coating MoS_2_ layer on the SnO_2_ thin film; this may support a high proportion of light to be refracted into it. A bilayer-coated film increases the imaginary part of the refractive index. This is essential in sensor response as a higher light absorption supports maximum light intensity variation. Additionally, the surface roughness of the proposed layers was studied. The results showed the roughness of SnO_2_ thin film and SnO_2_/MoS_2_ bilayer as 10 nm and 49 nm, respectively. The increased surface roughness of cladding material is one of the critical factors in enhancing the sensor response of the evanescent wave-based sensors [20,21]. 

## 5. Experimental Setup

**Figure 7:**
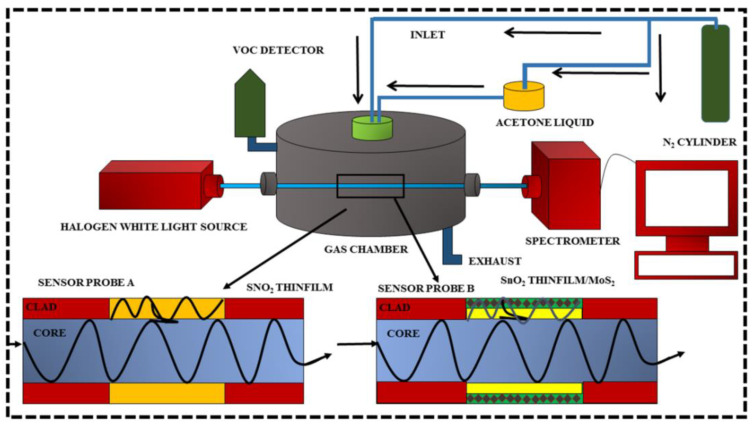
A fiber optic acetone sensor system was used in the experimental setup to evaluate the intensity variation for various concentrations ranging from 0 to 250 ppm.

The gas sensing setup of fiber optic-based sensor is shown in Figure 7. The prepared sensor probe is connected to a spectrometer (CCS200/M, Thorlabs Inc.) at one end and a halogen white light source (SLS201/M, Thorlabs Inc.) at the other. The spectrometer monitored the intensity fluctuation of acetone sensor probes prepared at regular time intervals. Acetone was combined with a carrier gas (N_2_) and introduced into the detecting chamber at various ppm levels (0–250 ppm). The carrier gas (N_2_) flow enters the chamber to saturate the starting intensity level after each measurement. The intensity variation at each concentration is used to determine the sensor response of the ready-made sensor probes. The experiment was conducted in a darkened space at a temperature of 27 °C.

### Acetone Sensing Mechanism

**Figure 8:**
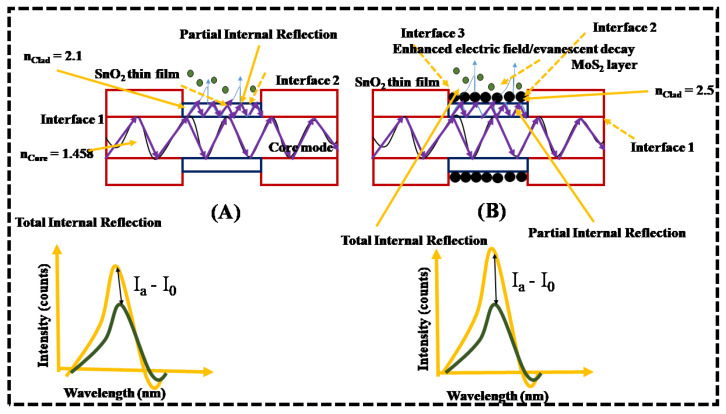
Schematic of the sensing mechanism of intensity loss of the proposed optical fiber-based sensors after the interaction of acetone concentration with the proposed (**A**) SnO_2_, and (**B**) SnO_2_/MoS_2_ sensing layers.

The proposed sensor probes comprise two different sensing layers: (1) SnO_2_ thin film layer, and (2) SnO_2_/MoS_2_ layer, with different etch rates of plastic clad layer. The schematic of probes with different clad thickness/different clad etch rates coated with SnO_2_ layer and SnO_2_/MoS_2_layer is shown in supplementary Figure S1. In SnO_2_-coated sensor probe, the sensing mechanism relies on the number of oxygen molecules adsorbed on the sensing layer, optical condition of the sensing layer and penetration depth of evanescent wave as shown in Figure 8. When SnO_2_ thin film is exposed to the air ambiance, oxygen molecules are adsorbed on the surface, resulting in the formation of oxygen ionic species, such as, $O^{-}$, $O_{2}^{-}$, and $O^{2-}$^.^ The adsorbed oxygen is temperature dependent, namely, $O_{2}^{-}$ when the temperature is below 100 °C, $O^{-}$ for temperatures 100–300 °C, and $O^{2-}$ for temperatures above 300 °C. In the present study, as the sensor operates at room temperature, O_2_^−^ is dominant. It is formed by trapping electrons from the conduction band since SnO_2_ is n type semiconductor with electrons (e^−^)as the majority carriers.

O_2_ (g) ↔ O_2_ (adsorb) (2)

O_2_ (adsorb) + e^−^ ↔ O_2_^−^ (3)

When the reducing CH_3_COCH_3_ gas molecules make contact with SnO_2_ surface, the previously adsorbed oxygen species readily interact with the organic gas molecules by releasing the trapped electrons (e^−^) back to the SnO_2_ conduction band. As a result of this electron donating nature of CH_3_COCH_3_, the conductivity of SnO_2_ rises, and this alters the optical properties of the layer. Hence, it is common for the adsorbed oxygen molecules on the sensor surface to have a dominant role in the regulating sensing process. The following is a possible proposed response mechanism [22]:

(4) $${\mathrm{CH}}_{3}{\mathrm{COCH}}_{3}\;(g)\;\to \;{\mathrm{CH}}_{3}{\mathrm{COCH}}_{3}\;(\mathrm{ad})$$

(5) $${\mathrm{CH}}_{3}{\mathrm{COCH}}_{3}\;(\mathrm{ad})+4O^{-}(\mathrm{ad})\;\to \;3{\mathrm{CO}}_{2}(g)+3H_{2}O+4e^{-}$$

This fluctuation in the optical characteristics of SnO_2_ layer affects the guided light inside the core of the optical fiber, resulting in intensity variation. Furthermore, the clad layer thickness and its refractive index condition affects the evanescent wave’s penetration depth, causing changes in the intensity of the reflected light inside the core. The primary objective of this study is to improve the sensing behaviour and make the probe more reactive to acetone molecules. As a result, in another set of sensor probes, a MoS_2_ layer is introduced over the SnO_2_ layer because it has a greater surface to volume ratio and provides better adsorption sites for nonpolar gas molecules.A similar detection mechanism occurs in the second set of sensor probes, and the additional layer leads to a larger surface to volume ratio.

**Figure 9:**
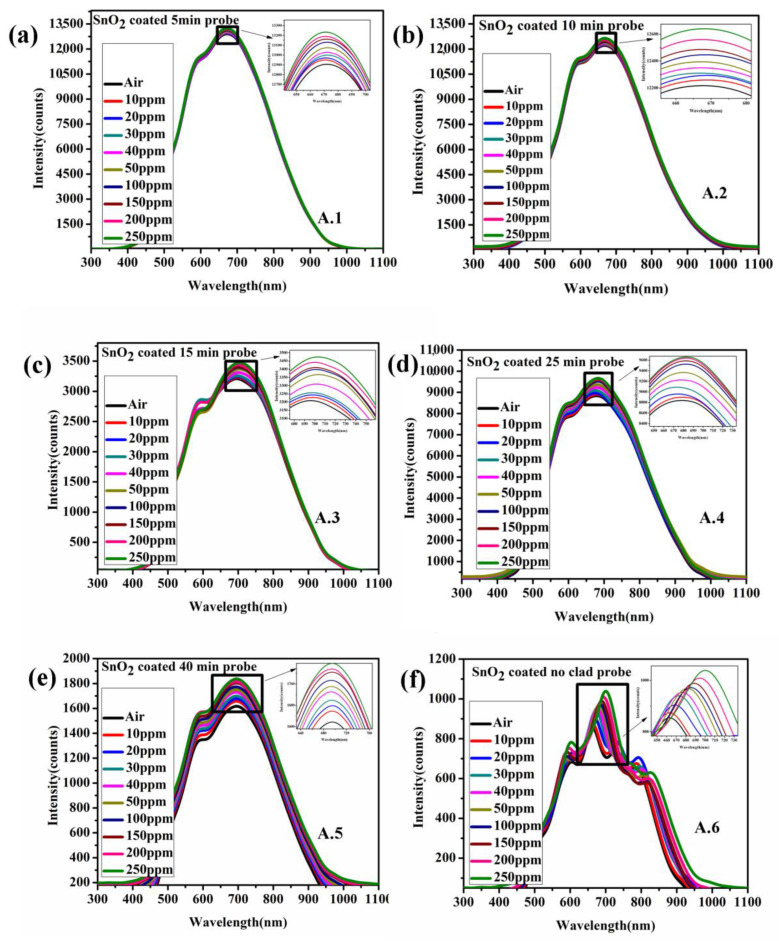
Spectral response of SnO_2_ thin film coated fiber optic acetone sensor with different clad etch probes (**a**) 5 min, (**b**) 10 min, (**c**) 15 min, (**d**) 25 min, (**e**) 40 min and (**f**) complete unclad.

**Figure 10:**
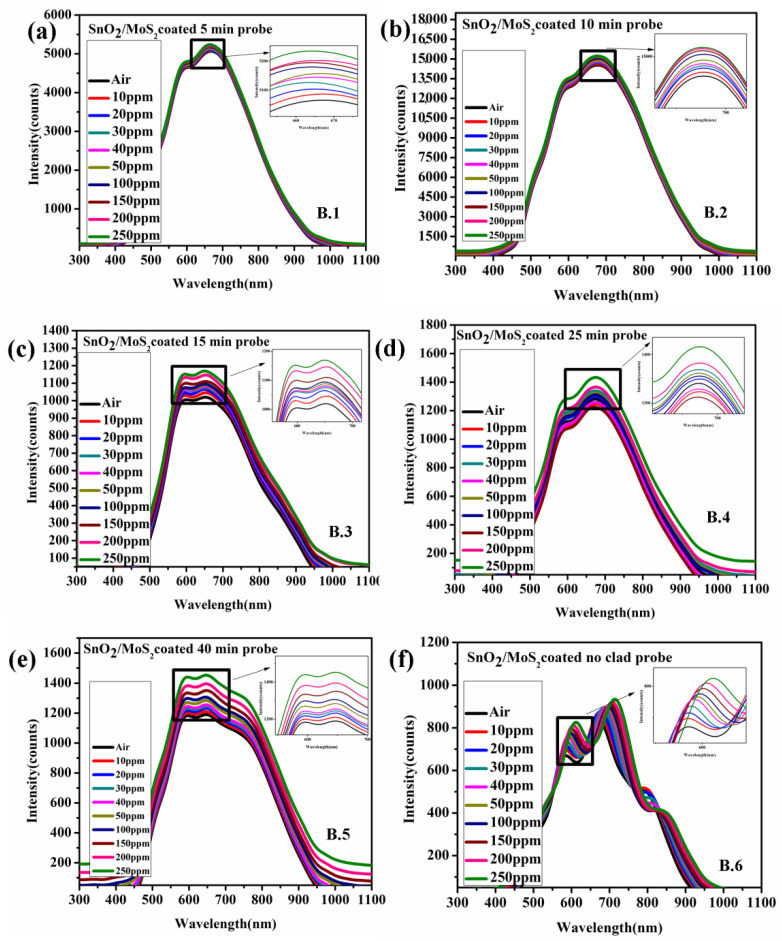
Spectral response of bilayer of SnO_2_ thin film/MoS_2_ layer coated fiber optic acetone sensor with different clad etch probes (**a**) 5 min, (**b**) 10 min, (**c**) 15 min, (**d**) 25 min, (**e**) 40 min and (**f**) complete unclad.

The proposed evanescent wave-based fiber optic sensor works on the principle of leaky mode condition, that is, n_cl_ > n_core_ and refractive index of the cladding is greater than the core (n_SnO2_ = ~1.7 and n_SnO2/MoS2_ = ~2.6). The partial internal reflection occurs inside the core, and light refracts into the SnO_2_ layer (interface 1). The refracted light in the SnO_2_ layer reaches interface 2 due to total internal reflection between the SnO_2_ thin film and air ambiance [23]. The proposed sensing layer can absorb light although it is a transparent material [24]. The evanescent wave at interface 2 reacts with the air/gas molecules. After significant changes, the light reflects to the core layer. The absorption of refracted rays and the evanescent field by air molecules differs from that of acetone molecules [25]. Hence, both these molecules affect the guiding light (core mode) inside the core differently and thus result in intensity modulation [14]. Similarly, in the MoS_2_/SnO_2_-coated probe, the change in refractive index of MoS_2_ occurs after the interaction of gas molecules. The electron transfer from acetone molecules to MoS_2_ results in a change in band gap energy and refractive index condition at interface 2, thereby affecting its evanescent field [15]. Additionally, the effects of clad etching play a significant role in the enhancement of sensor response. The entirely clad removed optical fiber favors a high amount of light interaction with the sensing layer, as shown in Figure 9 and Figure 10.

The spectral intensity characteristics of the proposed sensor probes SnO_2_ (Sensor probe A.(1–6)) and probes with a bilayer of SnO_2_/MoS_2_ (Sensor probe B.(1–6))-coated fiber optic sensor for acetone are shown in Figure 9a and Figure 10f. The peak intensity of the sensor probes is directly affected by changes in the refractive index of the sensor’s surface. The intensity variation of all sensor probes was studied between 0 and 250 ppm of acetone concentration. The intensity variation of sensor probes A.(1–6) grows linearly as the etch rate of the optical fiber increases. The penetration depth at the core/modified clad contact increases as the clad etch rate increases. The clad-removed sensor probe exhibits the most significant intensity fluctuation towards acetone concentration compared to the other sensor probes. Likewise, Set 2 sensor probe (bilayer of SnO_2_/MoS_2_) and the completely clad-removed sensor probe show maximum intensity variation compared to other sensor probes. Compared to SnO_2_ thin film, the change in intensity is foremost in the bilayer of the SnO_2_/MoS_2_-coated sensor probe due to enhanced electric field intensity at its interface [26]. Furthermore, the response of acetone molecules on the MoS_2_ layer is dependent on the electron transfer from acetone molecules to MoS_2_ particles, which results in a greater change in the band gap energy and refractive index condition. The bandgap energy and refractive index are inversely proportional, according to an empirical relationship developed by Herve and Vandamme [27]. To investigate this, the effective index of the SnO_2_/MoS_2_-coated film is measured using an ellipsometer. The acetone gas molecules treatment results in considerable variation in the refractive index of the SnO_2_/MoS_2_ coated glass substrate.The effective index variation of both SnO_2_, and SnO_2_/MoS_2_ coated substrate is shown in supplementary Figure S2. In addition, acetone molecules react with the MoS_2_ layer. Due to its non-homogenous condition and gaps among the particles, acetone molecules diffuse and possibly react with SnO_2_ thin film. In both the sensor probes, completely clad-removed optical fiber coated with SnO_2_ thin film and SnO_2_/MoS_2_ bilayer showed maximum intensity variation compared to other sensor probes. In the sensor probe B6, two peaks were observed, wherein the peak near 600 nm exhibits maximum intensity variation compared to other peaks. The bilayer of SnO_2_/MoS_2_ has a more significant impact on intensity variation than the SnO_2_ coated probe due to its higher absorption property (as confirmed in Section 4). In addition, the surface roughness of the bilayer SnO_2_/MoS_2_ is comparatively higher than the SnO_2_ thin film, resulting in maximum intensity loss of transmitted light inside the optical fiber. Along with intensity variation, the sensor probe B6 also reveals the wavelength shift due to its phase matching between core and leaky modes.

## 6. Sensor Response

The sensor response of the proposed sensor probes is defined as the change in intensity of air ambiance to the change in intensity of varying acetone concentrations [24].

(6) $$S_{r}=\frac{I_{0}-I_{g}}{I_{0}}\times 100\;\%$$

Here I_0_ is the intensity of air ambiance, and I_g_ is the intensity of acetone concentration. 

**Figure 11:**
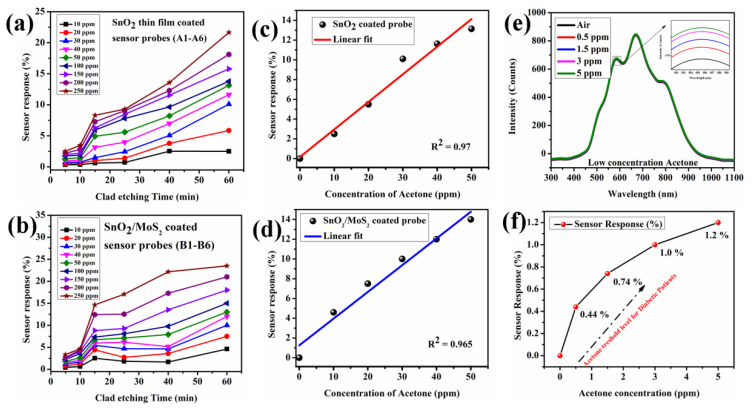
Sensor response of proposed sensor probes in terms of intensity variation (**a**) SnO_2_ thin film, (**b**) SnO_2_/MoS_2_-coated fiber optic acetone sensor with clad etch probes, and at all concentrations for (**c**) SnO_2_ thin film, (**d**) SnO_2_/MoS_2_. (**e**) Spectral response of the sensor probe B.6 for lower concentration of acetone, (**f**) Sensor response of the probe B.6 for lower concentration of acetone.

The sensor response of the proposed sensor probes ((A.1 to A.6) and (B.1 to B.6)) is shown in Figure 11. The result indicates that an increased clad etch rate enhances the sensor response for acetone concentrations between 0 and 250 ppm. The maximum sensor response at 250 ppm of acetone concentration for sensor probes A.1–A.6 are 2.5%, 3.47%, 8.3%, 9.31%, 13.58%, and 21.65%. The sensor response of all the concentrations is listed in Table 1. Similarly, the sensor probes B.1-B.6 show maximum sensor response of 3.34%, 4.67%, 14.66%, 17.04%, 22.14% and 23.5% for 250 ppm of acetone concentration (as listed in Table 2). The sensor response of the SnO_2_/MoS_2_-coated sensor probe is comparatively higher than SnO_2_ thin film coated probe due to possible gas interaction on both MoS_2_ and SnO_2_ layer.To analyze this, the MoS_2_ nanoparticle coated probe is tested with acetone concentration and its sensor response is~18.6 % (supplementary Figure S3). The sensor response of both the probes A.6 and B.6 were linear with acceptable R^2^ values of 0.97 and 0.965 in the range of 0–50 ppm, as shown in Figure 11c,d. Additionally, the maximum sensitive sensor probe B.6 is used to evaluate lower acetone concentrations, and the findings demonstrate that there is an intensity change of up to 0.5 ppm of acetone concentration, as shown in Figure 11e,f. Further, the limit of quantification of sensor probe B.6 is calculated as ~2.47 counts per ppm. As expected, the sensor response towards acetone is higher than in our previous work. The comparison with other sensors is listed in Table 3.

## 7. Response and Recovery

**Figure 12:**
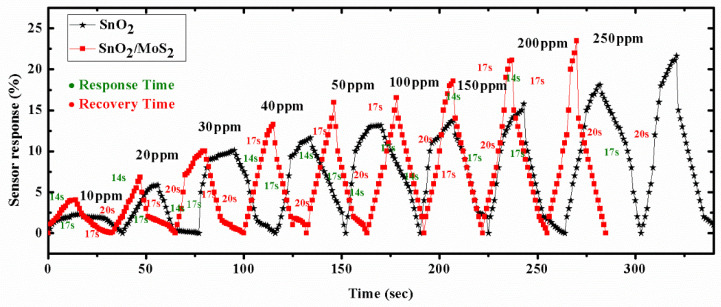
Dynamic response of proposed sensor probes A.6 and B.6 for measuring response and recovery time for acetone concentrations ranging from 0 to 250 ppm in terms of sensor response (intensity variation).

**Figure 13:**
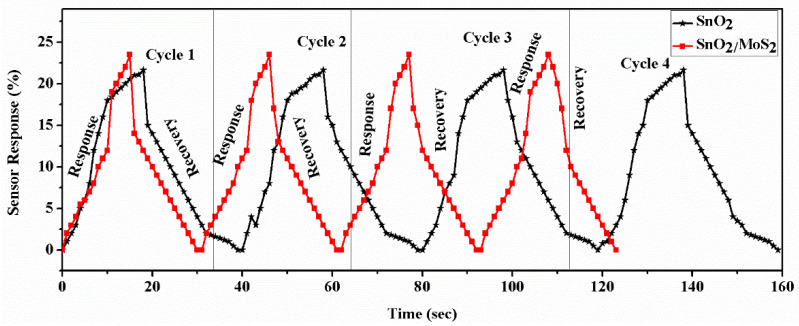
Dynamic response of proposed sensor probes A.6 and B.6 in terms of sensor response measurement and recovery in repeated four different cycles (intensity variation).

Using 250 ppm of acetone concentration for analysis, the response and recovery times for sensor probe B6 are displayed in Figure 12. The SnO_2_/MoS_2_-coated sensor is found to have a reaction time of 14 s and recovery time of 17 s. The SnO_2_/MoS_2_-coated sensor probe’s dynamic response time is also examined in different cycles. As shown in Figure 13, the sensor’s response time to different acetone concentrations ranges from 16 to 14 s. Additionally, the plot shows that the response and recovery times of SnO_2_ thin film coated sensor probes have changed noticeably in the third and fourth cycles. 

## 8. Selectivity

**Figure 14:**
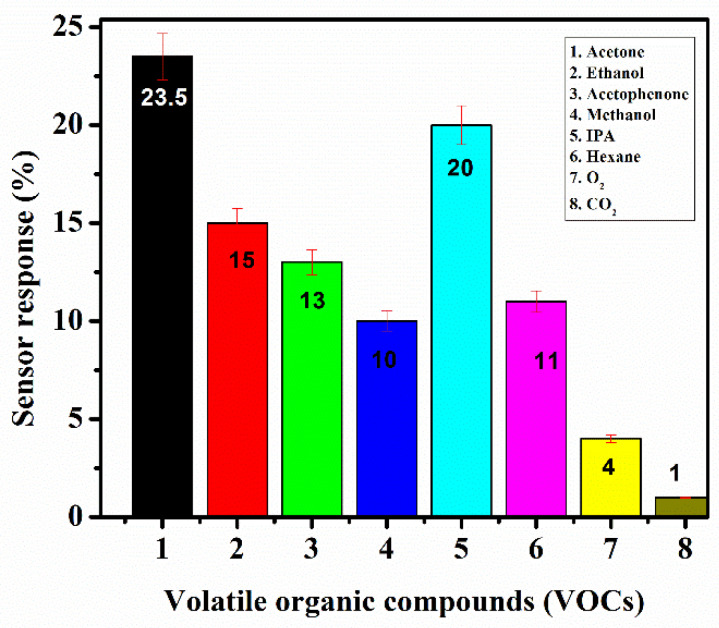
Selectivity of the proposed sensor probe B.6 was measured using the sensor response of different gas concentrations varying from 0 to 250 ppm passed into the sensing ambiance individually.

Selectivity is another crucial factor used to assess the detection efficiency of VOC sensors as exhaled breath comprises a variety of compounds. The selectivity for various VOCs at 250 ppm is displayed in Figure 14 to better explain the selective behaviour of SnO_2_/MoS_2_ at room temperature. The sensor response values for acetone, ethanol, acetophenone, methanol, IPA, hexane, oxygen, and carbon dioxide are about 23.5%, 15%, 13%, 10%, 20%, 11%, 4%, and 1%, respectively. The SnO_2_/MoS_2_-based sensor’s response to acetone was greater than its response to other gases, thereby demonstrating a significant level of selectivity.

## 9. Stability

**Figure 15:**
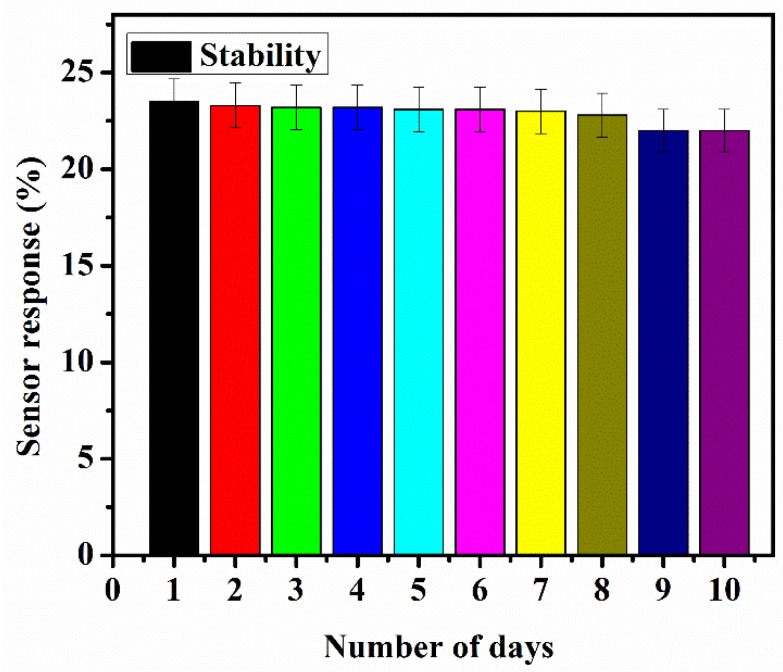
The stability of the suggested sensor probe B.6 (SnO_2_/MoS_2_) was examined by evaluating the sensor response to 250 ppm of acetone concentration every day for 10 consecutive days with a standard error of ~1.1.

The stability performance of the sensor probe B.6 (SnO_2_/MoS_2_) was analysed for ten days. The sensor probe B.6 is exposed to 250 ppm of acetone concentration for 45 min each day and its sensor response is calculated. As shown in Figure 15, the outcome indicated that the sensor probe B.6 is essentially stable for the intended duration of 10 days with a standard error of ~1.1.

## 10. Humidity Analysis

**Figure 16:**
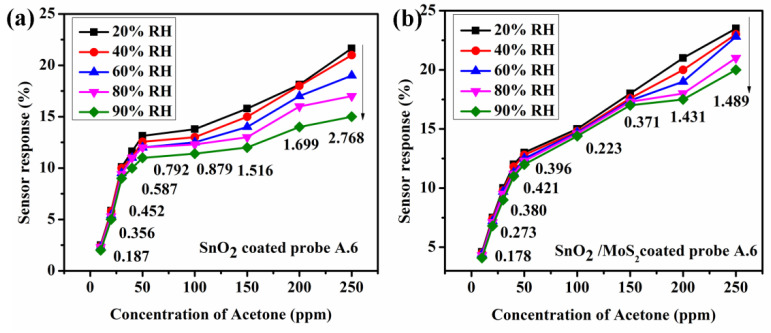
Variation of sensor response of the proposed sensor probes ((**a**) A.6 and (**b**) B.6) towards acetone concentrations was measured under different relative humidity ambiances.

According to E. Mansour et al. [33], the relative humidity (RH) in the exhaled breath ranged from 41.9% to 91.0% RH. To observe the influence of humidity, a water bubbler setup is linked to the sensor chamber’s input. To detect the relative humidity, a humidity sensor (DHT11) with an Arduino is attached to the chamber. As a result, the suggested sensor’s performance in terms of acetone concentration is examined under various conditions of humidity. The proposed sensor probes’ (A.6 and B.6) sensor response towards acetone concentration is studied. The results show that increasing the humidity level gradually decreases the sensor response of probes A.6 and B.6. Figure 16a,b depicts the computed standard deviation of change in sensor response. In contrast, the sensor probe B.6’s standard deviation is smaller because when RH rises, more H_2_O molecules are absorbed by both the MoS_2_ and SnO_2_ layers. The absorbed water molecules transfer a modest amount of charge from MoS_2_ to H_2_O [34]. As a result, the MoS_2_ layer’s effective refractive index fluctuates. As a result, the transmitted light changes more subtly than the other probe, therefore enhancing sensor responsiveness.

## 11. Prototype

**Figure 17:**
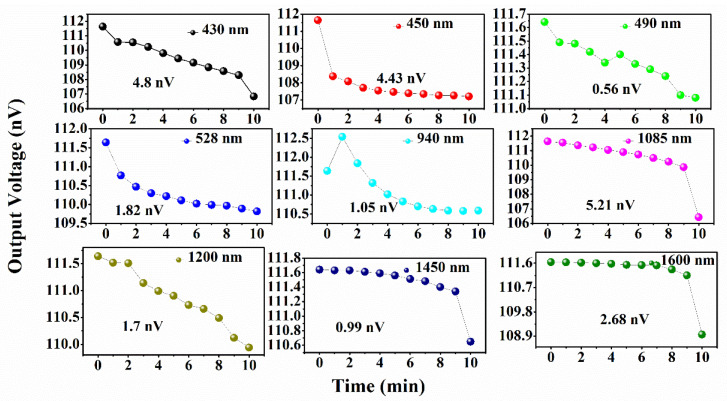
Output variation of the proposed sensor probe B.6 when exposed to 250 ppm IPA with different LED light source ranges (430 nm, 490 nm, 528 nm, 940 nm, 1085 nm, 1200 nm, 1450 nm and 1600 nm).

The prototype of the sensor probe is developed by miniaturizing the proposed sensor probe B.6 (SnO_2_/MoS_2_) and placing it inside a small-sized chamber. The sensor probe is connected with different ranges of light emitting diodes (namely, LED 430 nm, 450 nm, 490 nm, 528 nm, 940 nm, 1085 nm, 1200 nm, 1450 nm, and 1600 nm, Thorlabs Inc.) individually at one end and photodiode detector at the other end. The detailed prototype setup is shown in the supplementary Figure S4. The laser diode current controller is connected with the LED to supply a constant current of 40 mA. The output voltage is recorded using a photodiode amplifier connected to the detector. Acetone of 250 ppm concentration is passed into the sensing chamber, and output voltages are recorded for every 60 s up to 10 min. All the LED ranges showed a decrease in output voltage with time. The interaction of acetone molecules on the sensor surface reduces the intensity of light propagating inside the optical fiber core, resulting in output voltage drops. The output voltage drop for all LED ranges (Visible to IR) is shown in Figure 17.

## 12. Conclusions

Tin Oxide (SnO_2_) and bilayer of Tin Oxide/Molybdenum Disulfide (MoS_2_) based fiber optic sensors were investigated for acetone gas detection with different etch rates of plastic cladding. The characterisation analysis was carried out for the proposed sensing layers using X-ray diffraction, scanning electron microscope, ellipsometry, and UV spectroscopy. From XRD analysis, the prominent peak patterns of SnO_2_ indicate a polycrystalline structure with tetragonal phase. The diffraction peak of MoS_2_ implies hexagonal phase of MoS_2_. The structural and morphological analysis of SnO_2_ and SnO_2_/MoS_2_ were studied using SEM. All sensor probes were investigated with acetone concentrations varying from 0 to 250 ppm. The sensor response reveals that the completely clad-removed sensor probe (B.6) has a maximum sensor response of 23.5% towards acetone concentration of 250 ppm than other probes. Further, the response and recovery times of sensor probe B.6 were 14 s and 17 s, respectively. Moreover, other sensor parameters like stability and selectivity were studied. Additionally, the prototype of sensor probe B.6 was developed with different LED ranges, and the output voltage was recorded continuously for up to 10 min. The results revealed that LED 1085 nm (IR Range) showed a maximum voltage drop of 5.21 nV. Thus, sensor probe B.6 has the potential to detect lower concentrations of acetone in the field of diabetes monitoring.

## Figures and Tables

**Table 1:** Sensor response of SnO_2_ thin film coated fiber optic sensor for different concentrations of acetone.

Conc./Time	10 ppm	20 ppm	30 ppm	40 ppm	50 ppm	100 ppm	150 ppm	200 ppm	250 ppm
**5 min**	0.33%	0.47%	0.72%	0.94%	1.3%	1.74%	2.0%	2.2%	2.5%
**10 min**	0.36%	0.63%	0.77%	1.09%	1.46%	1.89%	2.21%	2.81%	3.47%
**15 min**	0.61%	1.01%	1.49%	3.14%	4.93%	5.92%	6.28%	7.28%	8.3%
**25 min**	0.71%	1.39%	2.44%	3.98%	5.59%	7.79%	8.48%	9.06%	9.31%
**40 min**	2.52%	3.79%	5.06%	6.98%	8.21%	9.65%	11.55%	12.3%	13.58%
**Complete clad removal**	2.5%	5.85%	10.1%	11.63%	13.14%	13.78%	15.79%	18.12%	21.65%

**Table 2:** Sensor response of SnO_2_ thin film/MoS_2_ coated fiber optic sensor for different acetone concentrations.

Conc./Time	10 ppm	20 ppm	30 ppm	40 ppm	50 ppm	100 ppm	150 ppm	200 ppm	250 ppm
**5 min**	0.41%	0.75%	1.2%	1.57%	1.82%	2.26%	2.56%	2.7%	3.34%
**10 min**	0.63%	1.29%	1.66%	2.0%	2.61%	3.55%	4.12%	4.4%	4.67%
**15 min**	2.52%	4.41%	5.44%	5.95%	6.73%	7.33%	8.8%	12.43%	14.66%
**25 min**	1.82%	2.73%	4.68%	6.15%	7.1%	8.07%	9.28%	11.53%	17.04%
**40 min**	1.67%	3.57%	4.63%	5.15%	7.91%	9.75%	13.52%	17.27%	22.14%
**Complete clad removal**	4.6%	7.5%	10%	12%	13%	15%	18%	21%	23.5%

**Table 3:** Comparison of sensor response of the proposed work with another sensor.

Sl.No	Sensing Principle	Sensing Layer	Sensor Response (%)	Limit of Detection	Concentration	Res./Rec.	Operating Temperature	Reference
1	Chemiresistive	TiO_2_	15.24	0.5 ppm	1000 ppm	10 s/9 s	270 °C	[28]
2	Chemiresistive	In_2_O_3_	8	1 ppm	50 ppm	32 s/38 s	260 °C	[29]
3	Chemiresistive	α-Ag_2_WO_4_ Nanorods	2.77	0.5 ppm	10 ppm	32 s/130 s	350 °C	[30]
4	Chemiresistive	Co_3_O_4_	7.6	10 ppm	100 ppm	25 s/5 s	180 °C	[31]
5	Evanescent Wave	SnO_2_	13.9	0.8 ppm	250 ppm	17 s/21 s	RT	[14]
6	Chemiresistive	Electrospun TiO_2_ nanorods	~21	1 ppm	500 ppm	14 s/8 s	RT	[32]
**7**	**Evanescent Wave**	**SnO_2_/MoS_2_**	**23.5**	**0.5 ppm**	**250 ppm**	**14 s/17 s**	**RT**	**Present work**

## Data Availability

Not Applicable.

## Supplementary Materials

The following supporting information can be downloaded at: https://www.mdpi.com/article/10.3390/bios12090734/s1, Figure S1: Schematic of Proposed sensor probes; Figure S2: Effective index of the sensing layers after acetone gas exposure (a) SnO_2_ layer, and (b) SnO_2_/MoS_2_ layer; Figure S3:MoS_2_ nanoparticle coated sensor probe; Figure S4: Prototype setup.
