# Supplementary material for: A Bilayer SnO2/MoS2-Coated Evanescent Wave Fiber Optic Sensor for Acetone Detection—An Experimental Study
Source: Biosensors (Basel). 2022 Sep 7;12(9):734. doi: 10.3390/bios12090734 (PMC9496449; doi:10.3390/bios12090734)
Supplement: Supplementary file 1 [file biosensors-12-00734-s001.zip › biosensors-1844292-supplementary.pdf]

biosensors

Supplementary

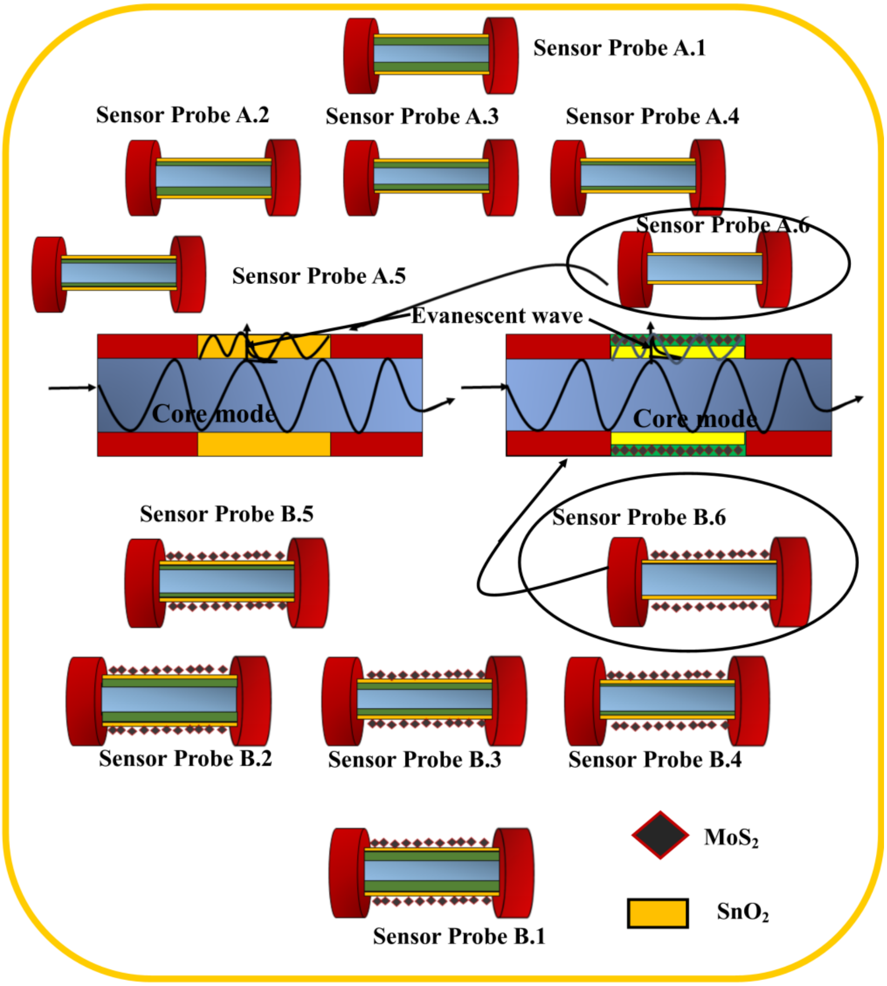

Figure S1. Schematic of proposed sensor probes.

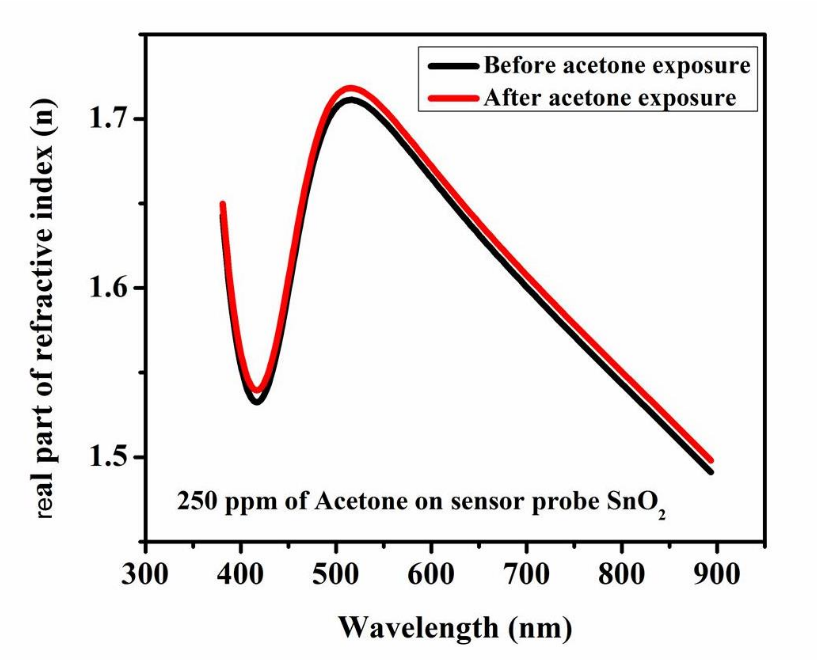

(a)

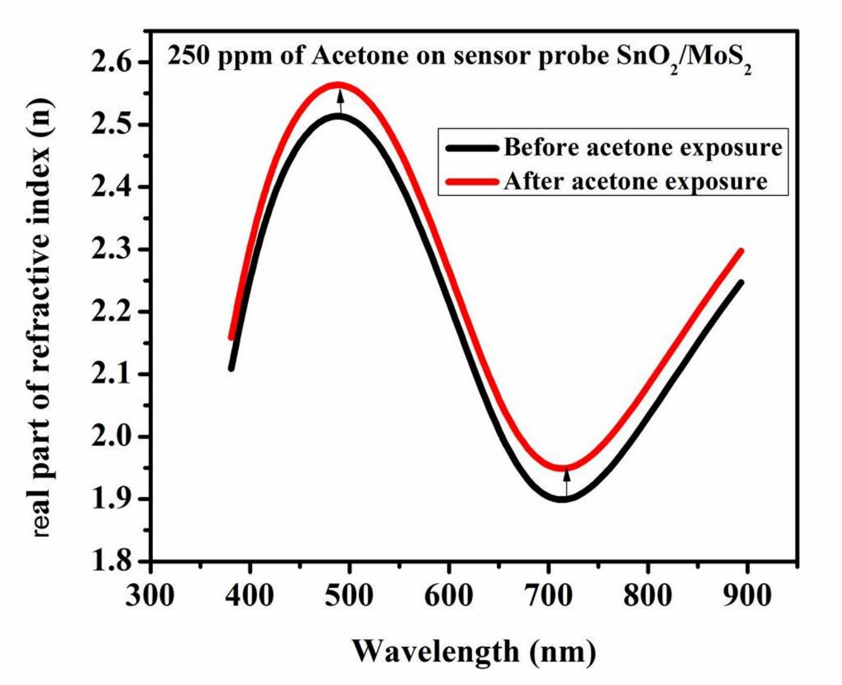

(b)

Figure S2. Effective index of the sensing layers after acetone gas exposure (a) SnO_2 layer, and (b) SnO_2/MoS_2 layer.

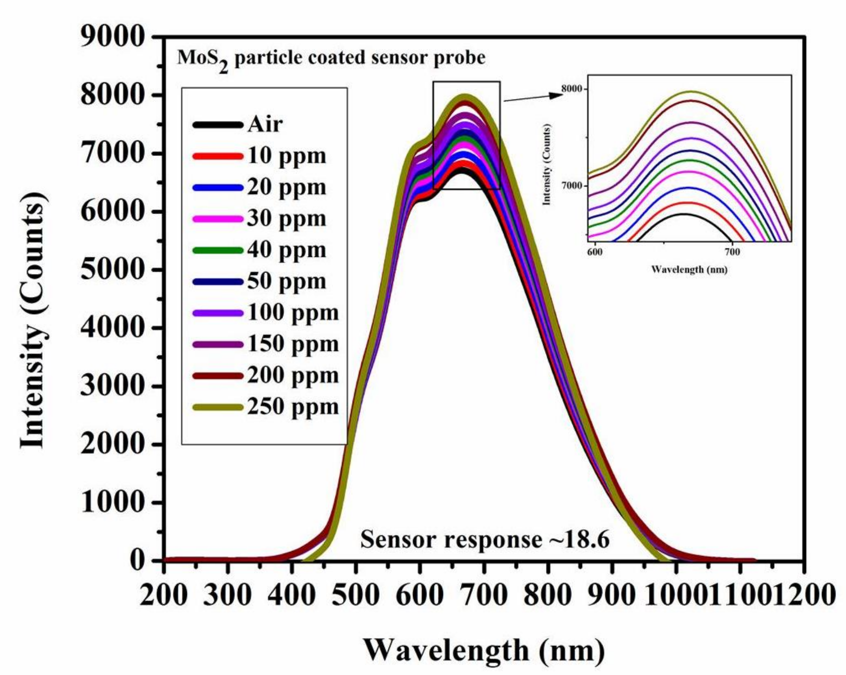

Figure S3. MoS_2 nanoparticle coated sensor probe.

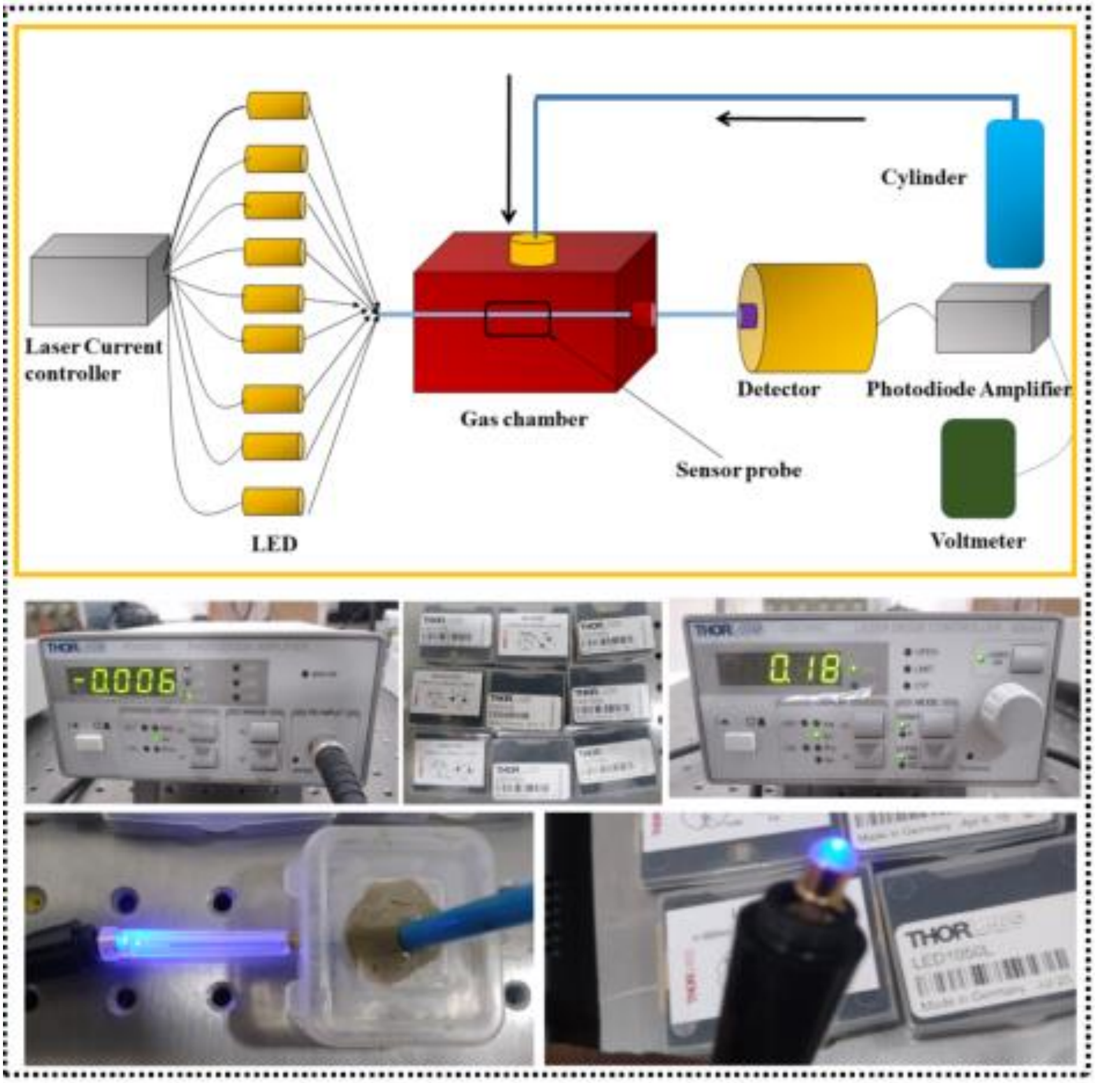

Figure S4. Prototype setup.
